# Both pre-frailty and frailty increase healthcare utilization and adverse health outcomes in patients with type 2 diabetes mellitus

**DOI:** 10.1186/s12933-018-0772-2

**Published:** 2018-09-27

**Authors:** Chia-Ter Chao, Jui Wang, Kuo-Liong Chien, COhort of GEriatric Nephrology in NTUH (COGENT) study group, Hung-Bin Tsai, Chih-Kang Chiang, Jenq-Wen Huang, Ding-Cheng Chan, Kuan-Yu Hung

**Affiliations:** 1 Department of Medicine, National Taiwan University Hospital BeiHu Branch, Taipei, Taiwan; 2 Nephrology Division, Department of Internal Medicine, National Taiwan University Hospital, Taipei, Taiwan; 3 Geriatric and Community Medicine Research Center, National Taiwan University Hospital BeiHu Branch, Taipei, Taiwan; 4 Institute of Epidemiology and Preventive Medicine, College of Public Health, National Taiwan University, No. 1, Sec. 4, Roosevelt Road, Taipei, 10617 Taiwan; 5 National Taiwan University Hospital (NTUH), Taipei, Taiwan

**Keywords:** Diabetes mellitus, Frail phenotype, Frailty, Hospitalization, Mortality

## Abstract

**Background:**

Diabetes mellitus (DM) correlates with accelerated aging and earlier appearance of geriatric phenotypes, including frailty. However, whether pre-frailty or frailty predicts greater healthcare utilization in diabetes patients is unclear.

**Methods:**

From the Longitudinal Cohort of Diabetes Patients in Taiwan (n = 840,000) between 2004 and 2010, we identified 560,795 patients with incident type 2 DM, categorized into patients without frailty, or with 1, 2 (pre-frail) and ≥ 3 frailty components, based on FRAIL scale (Fatigue, Resistance, Ambulation, Illness, and body weight Loss). We examined their long-term mortality, cardiovascular risk, all-cause hospitalization, and intensive care unit (ICU) admission.

**Results:**

Among all participants (56.4 ± 13.8 year-old, 46.1% female, and 84.8% community-dwelling), 77.8% (n = 436,521), 19.2% (n = 107,757), 2.7% (n = 15,101), and 0.3% (n = 1416) patients did not have or had 1, 2 (pre-frail), and ≥ 3 frailty components (frail), respectively, with Fatigue and Illness being the most common components. After 3.14 years of follow-up, 7.8% patients died, whereas 36.6% and 9.1% experienced hospitalization and ICU stay, respectively. Cox proportional hazard modeling discovered that patients with 1, 2 (pre-frail), and ≥ 3 frailty components (frail) had an increased risk of mortality (for 1, 2, and ≥ 3 components, hazard ratio [HR] 1.05, 1.13, and 1.25; 95% confidence interval [CI] 1.02–1.07, 1.08–1.17, and 1.15–1.36, respectively), cardiovascular events (HR 1.05, 1.15, and 1.13; 95% CI 1.02–1.07, 1.1–1.2, and 1.01–1.25, respectively), hospitalization (HR 1.06, 1.16, and 1.25; 95% CI 1.05–1.07, 1.14–1.19, and 1.18–1.33, respectively), and ICU admission (HR 1.05, 1.13, and 1.17; 95% CI 1.03–1.07, 1.08–1.14, and 1.06–1.28, respectively) compared to non-frail ones. Approximately 6–7% risk elevation in mortality and healthcare utilization was noted for every frailty component increase.

**Conclusion:**

Pre-frailty and frailty increased the risk of mortality and cardiovascular events, and entailed greater healthcare utilization in patients with type 2 DM.

**Electronic supplementary material:**

The online version of this article (10.1186/s12933-018-0772-2) contains supplementary material, which is available to authorized users.

## Background

Frailty, an emerging public health concern worldwide, is characterized by an age-related accumulation of health deficits accompanied with an increased susceptibility to exogenous and endogenous insults [1, 2]. Whether defined by the deficit-accumulation approach or the physical frailty approach, the presence of frailty correlates with functional impairment and adverse health outcomes, providing the background for subsequent disability, hospitalization, nursing home stay, and loss of independence [3, 4]. Current studies suggest that frailty is not synonymous with multimorbidity and contributes independently to impaired outcomes in old adults [4]. In addition, a transitory status termed “pre-frailty” has been proposed to mark the early and potentially reversible condition prior to full-fledged frailty; pre-frailty is reportedly associated with a significantly higher risk of cardiovascular diseases, prolonged hospital stay, and impaired quality of life in affected elderly [5, 6]. Researchers have proposed that the identification of pre-frailty can facilitate earlier and more aggressive intervention to contain the detrimental influences of ensuing frailty, disability, and possibly mortality [7]. In light of these results, understanding the effect of frailty and especially pre-frailty, on the overall health and patients’ healthcare utilization assumes great importance for the care of old patients.

The global burden of diabetes mellitus (DM) rises with ageing, and is accompanied by a high mortality among affected individuals [8]. In Taiwan, a nationwide survey found that the overall incidence of DM rose from 0.76 to 0.93% within 10 years, equivalent to a 25% increase, and there were 1.22 million Taiwanese affected by DM in 2009. This was accompanied by an 80% increase in the total diabetic population and 55% increase in the prevalence rate during the sampling period [9]. Patients aged 60 or higher accounted for the majority (30–50%) of the diabetic population, and the proportion of male outnumbered that of female after 2005 [9]. On the other hand, the annual incidence of type 1 DM remained stable, with an increased risk among female children of older age [10].

Frailty is recognized as an influential complication for patients with DM, irrespective of age. In a large group of middle-aged adults, Chode et al. found that individuals with diabetes had a higher likelihood of being frail than those without diabetes, and the presence of frailty in DM patients conferred an even greater risk of functional impairment and deteriorated performance [11]. Another group also revealed that frailty in middle-aged to older diabetes adults predisposed these patients to limitations in activities of daily living (ADLs) compared to non-frail ones [12]. The pathogenic linkage between DM and frailty potentially includes premature senescence of organ systems in a hyperglycemic status, chronic inflammation, increased oxidative stress, advanced glycation end-product accumulation, and insulin resistance, although a conclusive list is still under active pursuit [13, 14]. Nonetheless, previous data have already established the prognostic importance of frailty in patients with diabetes [15], while only a few studies have addressed the significance of pre-frailty. Furthermore, most studies focused on the adverse health outcomes of frailty including overall survival and functional outcomes, but the issue of healthcare utilization is under-recognized, especially among patients with diabetes. We hypothesized that both pre-frailty and frailty increased healthcare utilization in addition to mortality in patients with type 2 DM. We aimed to examine whether diabetic patients with pre-frailty or frailty consumed more healthcare resources than those without, using a national administrative database from Taiwan, an area with universal health coverage and a suitable substrate for analysis.

## Methods

### Ethical approval

The institutional review board of National Taiwan University Hospital (No. 201802063W) approved the current study; the study protocol adheres to the declaration of Helsinki. Informed consent was waived due to data anonymity.

### Participant enrollment and data sources

**Fig. 1:**
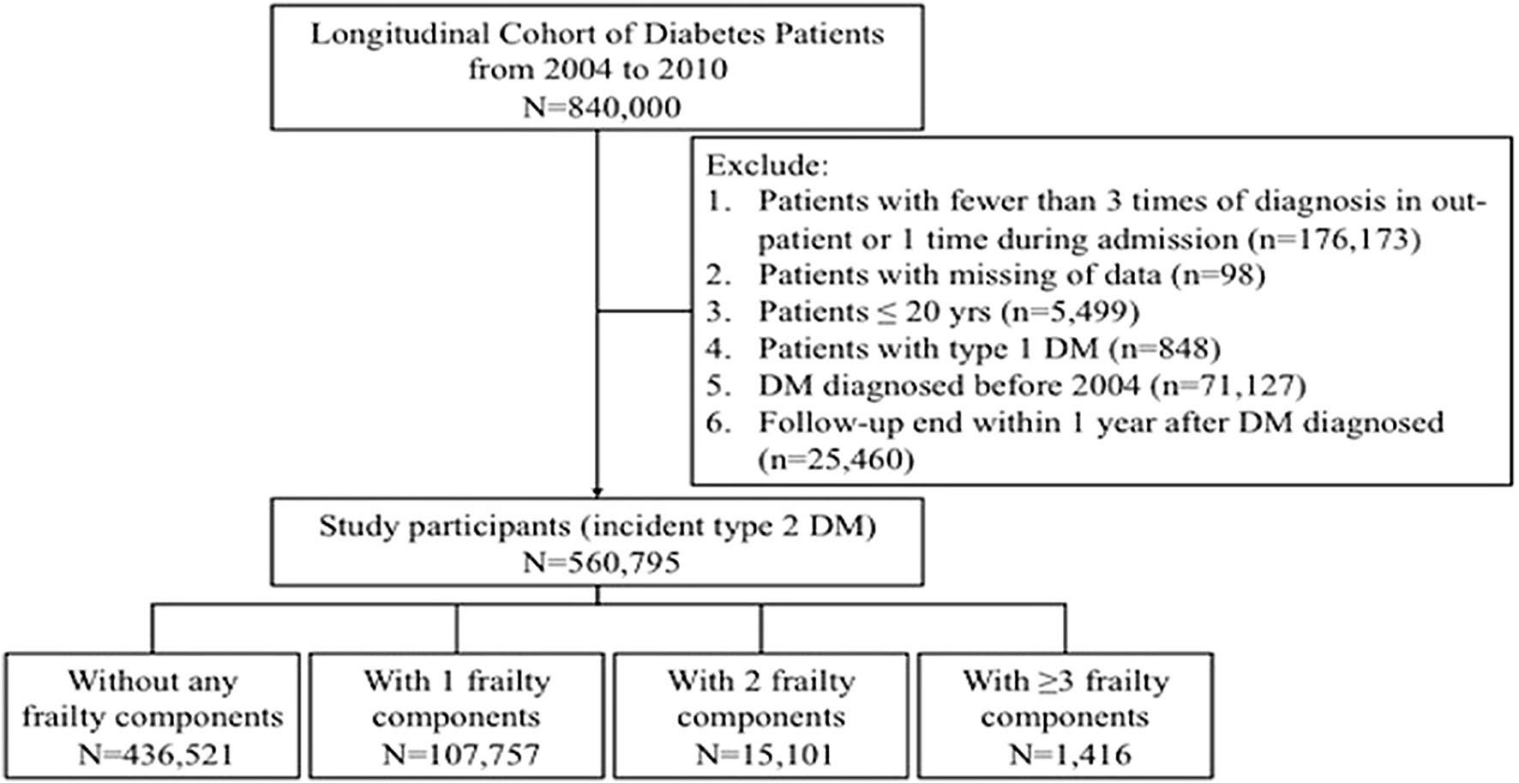
Flow chart of candidate selection in the current study. *DM* diabetes mellitus

We harnessed the Longitudinal Cohort of Diabetes Patients database (LHDB), a population-based data source compiled by the National Health Research Institute of Taiwan, between 2004 and 2010, for participant recruitment. The database consists of an annual random sampling of 120,000 patients with an incident DM diagnosis from all areas of Taiwan, with their clinical records retrospectively traced up to 1999 and followed until 2014 [16], constituting a longitudinal cohort for epidemiologic analysis. The diagnosis of DM was established based on the presence of at least two times of out-patient International Classification of Disease-9-Clinical Modification (ICD-9-CM) codes 250.xx within 1 year, at least one time of DM diagnosis accompanied by prescriptions of any oral anti-diabetic agents (OADs), or at least one diagnosis during admission. Prior studies have affirmed of the validity and the utility of this diabetes cohort for analyzing short- and long-term outcome predictors and the quality of care among DM patients [16, 17]. We further narrowed down the diagnostic criteria by increasing the times of diagnosis required to at least three, and excluded pediatric patients (≤ 20 years), patients with type 1 DM, prevalent DM patients, and patients with premature mortality after developing type 2 DM (Fig. 1). The date when patients satisfied the criteria for DM diagnosis (the last out-patient clinic with a DM diagnosis) was designated as the index date. The severity of DM was evaluated using adjusted diabetic complication severity index (aDCSI) [18–20]. In brief, aDCSI is a modified version of DCSI and gauges the severity of DM based on the number of diabetic complications without laboratory finding criteria. Results from aDCSI exhibit good correlation with the original DCSI ones, and higher aDCSI scores are associated with a greater number of hospitalizations among DM patients during follow-up [18].

### Exposures and primary outcomes

The primary exposure variable in this study was the presence and the severity of frailty. There are two approaches for assessing frailty in the existing literature, frail index and frail phenotype, both of which exhibit close associations with adverse outcomes in diverse populations [1, 2]. Evaluation of frailty severity was performed with the FRAIL scale, a highly cited and well-validated instrument consisting of five components (Fatigue, Resistance, Ambulation, Illness, and Loss of weight) [21–23]. We chose the FRAIL scale since its applicability in DM patients has been affirmed in the literature [11, 24]. However, the FRAIL scale is often assessed in research context but rarely during clinical encounter, rendering large-scale frailty screening based on FRAIL scale difficult [25]. Consequently, we attempted to operationalize frailty using FRAIL scale through the formulation of different diagnostic groupings.

Each frailty component was assessed based on the presence of selective diagnoses during at least two outpatient clinics or one hospitalization outlined in Table 1, within the preceding 5 years of the index date. Because the claim database did not contain measured clinical data (laboratory data), or results from in-person interviews or physical examinations, we selected diagnoses based on keyword search and relevant literature review [26, 27], followed by an in-depth review of expert geriatricians and epidemiologists, with consensus reached after two rounds of discussions. Diagnostic codes with close proximity or as surrogates to the underlying nature of the frailty components were included in the list (Table 1). The “Fatigue” component was identified using diagnoses containing the keywords “malaise” or “asthenia”. The ICD-9-CM code 780.7x (malaise and fatigue) has been shown to effectively capture patient complaint by others and in domestic studies [28, 29]. We additionally include 300.5 (neurasthenia), 797 (senile asthenia), and 780.99 (general weakness) to increase the detection efficiency and avoid under-reporting.

**Table 1:** Diagnostic groupings used to identify cases with pre-frailty and frailty

FRAIL scale components	ICD-9-CM codes
Fatigue	300.5, 780.7x, 797, 780.99
Resistance	799.3, E880, E881, E882, E883, E884, E885, E888
Ambulation	719.7, 781.2
Illnesses	HTN (401.x–405.x), Cancer (140.x–208.x), COPD (491.x), AMI (410.x), CHF (428.x), angina (411.1, 411.81, 411.89, 413.0, 413.1, 413.9), asthma (493.x), arthritis (99.3, 274, 696.0, 695.4, 710.x, 711.x, 712.x, 714.x, 713.x, 715.x, 716.x, 719.x, 720.x, 721.x), stroke (430.x–438.x), CKD (016.0x, 042.x, 095.4, 189.x, 223.x, 236.9x, 250.4x, 271.4, 274.1x, 403.x, 404.x, 440.1, 442.1, 446.21, 447.3x, 572.4, 580.x–589.x, 590.x, 591.x, 593.x, 642.1x, 646.2x, 753.x, 984.x)
Body weight loss	260, 261, 262, 263.0, 263.1, 263.8, 263.9, 728.2, 780.94, 783.21, 783.3, 783.7, 799.4

For “Resistance”, patients need to have difficulty stair climbing, and in this study, we identified “Resistance” based on the presence of “debility” or “fall”. Stair negotiation limitation is an important surrogate for functional decline and debility, while prior studies established that the code 799.3 could identify those with physical deconditioning [28, 30] and a tendency for fall. A history of fall correlates closely with gait imbalance and difficulty in stair climbing [31]. In light of these, we believe that the selected code groups can identify those with difficulty in stair climbing. “Ambulation” was recognized based on “walking difficulty” or “gait abnormality”. The diagnostic codes 719.7 (difficulty in walking) and 781.2 (gait abnormality) have been utilized to identify those with ambulation difficulty in large claim databases [28]. “Illness” was coded using the presence of at least four morbidities out of totally 10 within the lists (Table 1). The diagnostic code combinations for identifying each chronic illness have been validated by others in the literature and also in the database we used [32–34]. “Loss of weight” was identified using codes associated with “malnutrition”, “feeding difficulty”, “cachexia”, or “muscle wasting”, an approach validated in the past [32]. We defined pre-frailty as the presence of 1 or 2 components described above, and frailty as the presence of more than 2 components [21, 35].

The primary outcome of our study was the use of healthcare resources, including the incidence of hospitalization or intensive care unit (ICU) stay during follow-up. Secondary outcomes included the overall mortality and incident cardiovascular events during follow-up. Cardiovascular events examined in this study included incident myocardial infarction, unstable angina, heart failure, new-inset stroke (ischemic, hemorrhagic, or transient ischemic attack), and submission to revascularization procedures (coronary artery bypass graft/percutaneous coronary intervention) [36]. For patients with cardiovascular events, hospitalization or ICU admission, only the first episode of the event was included in our analysis.

### Statistical analysis

Continuous and categorical variables were compared using independent t-tests and Chi square tests, respectively, while comparisons of more than two groups were performed with ANOVA. All statistical analyses were performed using SAS software (SAS institute, Cary, NC, USA), and two-tail P-values lower than 0.05 were considered significant.

We first examined the distribution of each frailty component in the entire diabetes cohort, followed by categorization of patients with type 2 diabetes based on their frail component counts (0, 1, 2, ≥ 3) or the presence or absence of pre-frailty or frailty. We compared the demographic profiles, comorbidity statuses, DM severity, selected co-existing medications with influences on the survival and functional status, and OAD types between patients without and with different severities of frailty. We followed these patients until the end of this study or until 12/31/2010, and constructed Kaplan–Meier survival and event-free curves with between-group comparisons with a log-rank test. Cox proportional hazard modeling with mortality, cardiovascular events, hospitalization, and ICU admission as dependent variables was conducted, incorporating demographic (age, gender) and comorbidity profiles (hypertension, hyperlipidemia, chronic liver disease, chronic obstructive pulmonary disease, chronic kidney disease, atrial fibrillation, coronary artery disease, cerebrovascular disease, peripheral vascular disease, malignancy, gout, osteoarthritis, osteoporosis, obesity, and mental illnesses), experiences of hypoglycemia, substance use history (smoking and alcohol abuse), aDCSI, and co-existing medications (aspirin, β-blocker, angiotensin converting enzyme inhibitor, angiotensin receptor blocker, clopidogrel, statin, fibrate, allopurinol, warfarin, benzodiazepine, anti-depressants, anti-psychotics, and all OADs identified) in full models. We also constructed Cox proportional hazard-based survival curves based on frailty component counts. Finally, we evaluated the contribution of each frailty component to the outcomes of interest individually, based on Cox regression modeling. Sensitivity analyses were also conducted to examine the validity of our findings.

## Results

Between 2004 and 2010, 840,000 patients with incident diabetes were identified; after using stricter criteria for DM diagnosis and excluding prevalent diabetes patients and pediatric and type 1 DM patients, 560,795 incident type 2 DM patients were finally enrolled for further analysis (Fig. 1). Among these patients, the mean age was 56.4 ± 13.8 years, with 46.1% female and 84.8% being community-dwelling. 77.8% (n = 436,521), 19.2% (n = 107,757), 2.7% (n = 15,101), and 0.3% (n = 1416) did not have or had 1, 2, and ≥ 3 frailty components, respectively, yielding a prevalence of pre-frailty and frailty at 21.9% and 0.3%, respectively. Among participants with diabetes with pre-frailty or frailty, Fatigue was the most common component qualified (60–95%), followed by Illness (30–95%) and Body weight loss (3–55%) (Table 2). Ambulation was the least common qualified component.

**Table 2:** Distribution of frailty components among study participants

Frailty components	No frailty (n = 436,521)	Pre-frailty		Frailty
		1 component (n = 107,757)	2 components (n = 15,101)	≥ 3 components (n = 1416)
Fatigue	0 (0)	66,035 (61.3)	12,666 (83.9)	1290 (91.1)
Resistance	0 (0)	2513 (2.3)	1696 (11.2)	529 (37.4)
Ambulation	0 (0)	1337 (1.2)	1076 (7.1)	425 (30)
Illnesses	0 (0)	34,311 (31.8)	12,541 (83.1)	1350 (95.3)
Body weight loss	0 (0)	3561 (3.3)	2223 (14.7)	738 (52.1)

Severity of frailty was positively correlated with age, severity of diabetes and the incidence of most comorbidities except hyperlipidemia (Table 3). People with higher severity of frailty were more likely to receive medications that potentially affect their overall and cardiovascular survival, except statin and fibrate (Table 3). In contrast, those with higher frailty severity, possibly due to their increased risk of hypoglycemia, were less likely to receive multiple OADs including biguanides, sulfonylurea, α-glucosidase inhibitors, thiazolidinediones, and dipeptidyl peptidase 4 inhibitors (Table 3).

**Table 3:** Baseline characteristics of diabetic participants with different severities of frailty

Variables	No frailty (n = 436,521)	Pre-frail		Frailty	*p* value
		1 component (n = 107,757)	2 components (n = 15,101)	≥ 3 components (n = 1416)	
Demographic profiles					
Age (years)	54.8 ± 13.2	60.8 ± 14.3	69.4 ± 12.7	75.1 ± 11.2	< *0.01*
Female (%)	197,022 (45.1)	53,089 (49.3)	7631 (50.5)	685 (48.4)	< *0.01*
Severity of diabetic complications					
aDCSI scores	0.2 ± 0.6	0.5 ± 1	1 ± 1.3	1.4 ± 1.5	< *0.01*
Comorbidity profiles					
Charlson comorbidity index	1.7 ± 1.4	2.8 ± 2.1	4.4 ± 2.3	5.4 ± 2.4	< *0.01*
Hypertension (%)	221,259 (50.7)	70,979 (65.9)	13,259 (87.8)	1299 (91.7)	< *0.01*
Hyperlipidemia (%)	179,826 (41.2)	48,416 (44.9)	6606 (43.8)	452 (31.9)	< *0.01*
Chronic liver disease (%)	96,954 (22.2)	34,510 (32)	5426 (35.9)	534 (37.7)	< *0.01*
COPD (%)	19,644 (4.5)	20,574 (19.1)	6594 (43.7)	839 (59.3)	< *0.01*
Chronic kidney disease (%)	45,991 (10.5)	26,097 (24.2)	6719 (44.5)	787 (55.6)	< *0.01*
Atrial fibrillation (%)	25,863 (5.9)	15,794 (14.7)	4293 (28.4)	533 (37.6)	< *0.01*
Prior coronary atherosclerosis (%)	52,211 (12)	29,372 (27.3)	7489 (49.6)	825 (58.3)	< *0.01*
Cerebrovascular disease (%)	35,748 (8.2)	25,100 (23.3)	7459 (49.4)	970 (68.5)	< *0.01*
Peripheral vascular disease (%)	5673 (1.3)	3101 (2.9)	855 (5.7)	99 (7)	< *0.01*
Malignancy (%)	17,791 (4.1)	9713 (9)	2451 (16.2)	260 (18.4)	< *0.01*
Gout (%)	58,414 (13.4)	22,134 (20.5)	4189 (27.7)	385 (27.2)	< *0.01*
Osteoarthritis (any site) (%)	77,694 (17.8)	36,908 (34.3)	8183 (54.2)	921 (65)	< *0.01*
Osteoporosis (%)	20,543 (4.7)	11,277 (10.5)	2962 (19.6)	376 (26.6)	< *0.01*
Obesity (%)	7051 (1.6)	1626 (1.5)	198 (1.3)	12 (0.9)	< *0.01*
Mental illnesses (%)	52,794 (12.1)	24,302 (22.6)	5329 (35.3)	567 (40)	< *0.01*
Prior hypoglycemia (%)	461 (0.1)	350 (0.3)	136 (0.9)	17 (1.2)	< *0.01*
Substance use history					
Smoking (%)	2761 (0.6)	948 (0.9)	151 (1)	9 (0.6)	< *0.01*
Alcoholism (%)	3679 (0.8)	1423 (1.3)	240 (1.6)	19 (1.3)	< *0.01*
Medications with potential influences on functional status					
Aspirin (%)	128,686 (29.5)	51,704 (48)	10,760 (71.3)	1102 (77.8)	< *0.01*
β-blocker (%)	193,727 (44.4)	65,833 (61.1)	11,624 (77)	1069 (75.5)	< *0.01*
ACEI (%)	126,276 (28.9)	45,194 (41.9)	9529 (61.3)	873 (61.7)	< *0.01*
ARB (%)	112,080 (25.7)	38,508 (35.7)	7778 (51.5)	695 (49.1)	< *0.01*
Clopidogrel (%)	11,304 (2.6)	7875 (7.3)	2081 (13.8)	192 (13.6)	< *0.01*
Statin (%)	142,839 (32.7)	39,143 (36.3)	5730 (37.9)	417 (29.5)	< *0.01*
Fibrate (%)	68,427 (15.7)	20,376 (18.9)	3080 (20.4)	196 (13.8)	< *0.01*
Allopurinol (%)	13,087 (3)	5914 (5.49)	1311 (8.7)	115 (8.1)	< *0.01*
Warfarin (%)	4708 (1.1)	3286 (3.1)	844 (5.6)	87 (6.1)	< *0.01*
Benzodiazepine (any) (%)	250,841 (57.5)	83,345 (77.4)	13,542 (89.7)	1278 (90.3)	< *0.01*
Anti-depressants (%)	72,752 (16.7)	32,912 (30.5)	7028 (46.5)	767 (54.2)	< *0.01*
Anti-psychotics (%)	110,884 (25.4)	43,810 (40.7)	8769 (58.1)	960 (67.8)	< *0.01*
Oral anti-diabetic agents/Insulin					
Biguanides (%)	220,362 (50.5)	46,116 (42.8)	5327 (35.3)	344 (24.3)	< *0.01*
Sulfonylurea (%)	204,599 (46.9)	43,179 (40.1)	5005 (33.1)	339 (23.9)	< *0.01*
Meglitinides (%)	21,637 (5)	5719 (5.3)	940 (6.2)	63 (4.5)	< *0.01*
α-Glucosidase inhibitors (%)	27,784 (6.4)	7028 (6.5)	920 (6.1)	51 (3.6)	< *0.01*
TZD (%)	18,442 (4.2)	3530 (3.3)	392 (2.6)	28 (2)	< *0.01*
DPP4 inhibitors (%)	5810 (1.3)	1141 (1.1)	119 (0.8)	7 (0.5)	< *0.01*
Insulin (%)	21,148 (4.8)	6073 (5.6)	1112 (7.4)	109 (7.7)	< *0.01*

*ACEI* angiotensin-converting enzyme inhibitor, *aDCSI* adapted diabetes complications severity index, *ARB* angiotensin receptor blocker, *COPD* chronic obstructive pulmonary disease, *DPP4* dipeptidyl peptidase 4, *TZD* thiazolidinedione

**Fig. 2:**
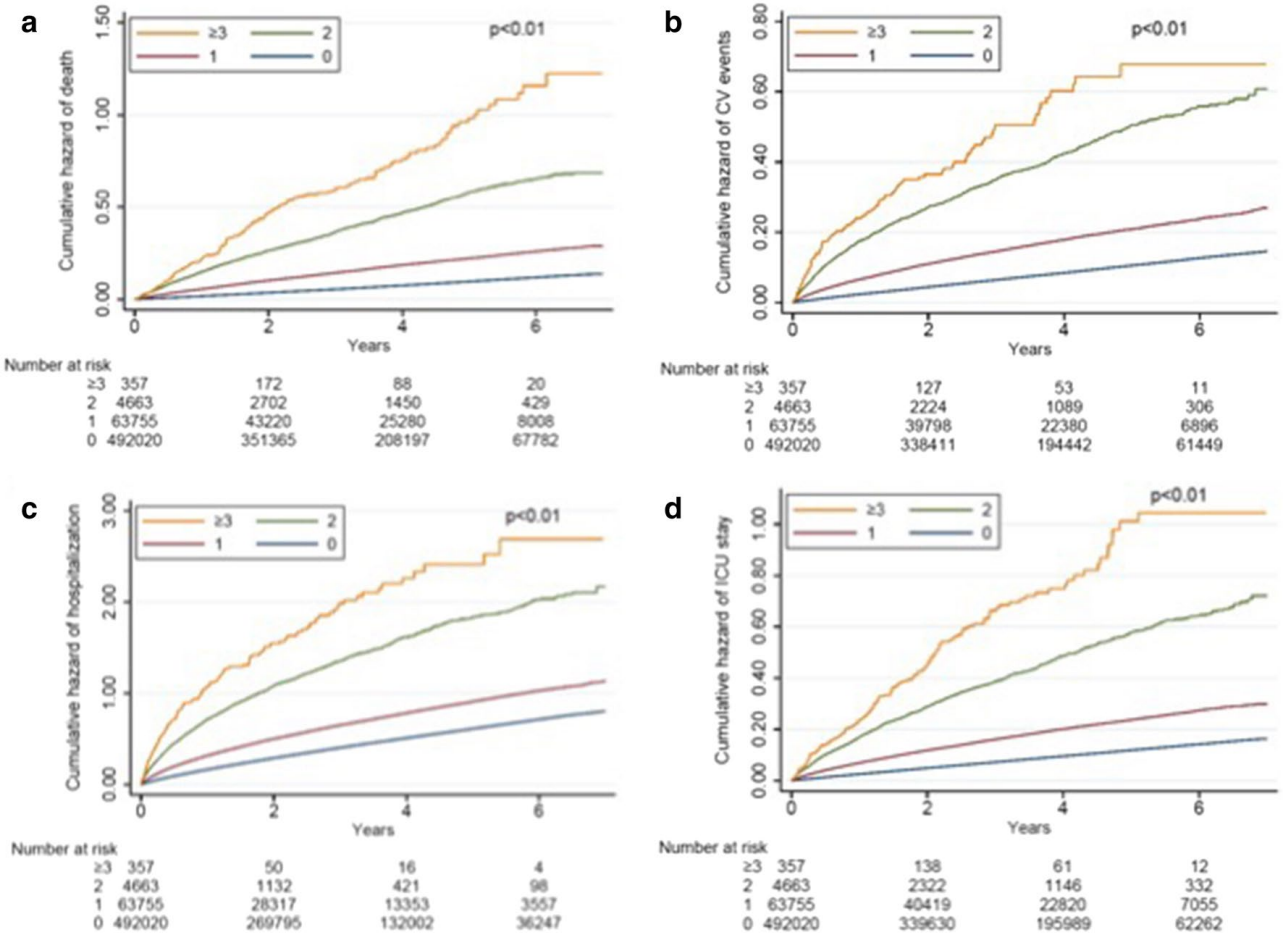
**a** Overall survival, **b** CV event-free, **c** hospitalization-free, and **d** ICU admission-free curves of the enrolled diabetes participants, based on Kaplan–Meier technique. *CV* cardiovascular, *ICU* intensive care unit

**Fig. 3:**
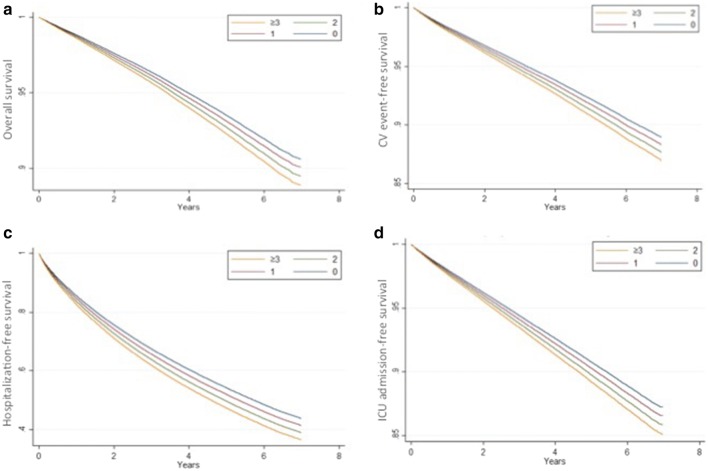
**a** Overall survival, **b** CV event-free, **c** hospitalization-free, and **d** ICU admission-free curves of the enrolled diabetes participants, based on Cox proportional hazard modeling. *CV* cardiovascular, *ICU* intensive care unit

After a mean 3.14 years of follow-up, mortality rate was 7.8%, and cardiovascular event rate was 8.1%. In addition, 36.6% and 9.1% of patients had an episode of hospitalization and ICU admission, respectively. Kaplan–Meier survival curves demonstrated that pre-frail and frail diabetes participants showed significantly higher mortality, cardiovascular risk, and healthcare utilization (hospitalization and ICU admission) than non-frail individuals (Fig. 2; P < 0.001 for comparisons between all four groups for all endpoints). Cox proportional hazard modeling revealed that compared to non-frail diabetes participants, diabetes participants with 1, 2, or ≥ 3 frailty components had gradually higher overall mortality (for 1, 2, and ≥ 3 components, hazard ratio [HR] 1.05, 95% confidence interval [CI] 1.02–1.07; HR 1.13, 95% CI 1.08–1.17; and HR 1.25, 95% CI 1.15–1.36, respectively) after adjusting for demographic profiles, comorbidities, diabetes severity, and medications (Table 4). For every 1-component increase, 6% higher mortality was noted (HR 1.06; 95% CI 1.04–1.08). Similarly, compared to those without frailty, diabetes participants with 1, 2, or ≥ 3 frailty components had higher risk of developing cardiovascular events (for 1, 2, and ≥ 3 components, HR 1.05, 95% CI 1.02–1.07; HR 1.15, 95% CI 1.1–1.2; and HR 1.13, 95% CI 1.01–1.25, respectively), with a 6% higher cardiovascular risk per component increase (HR 1.06, 95% CI 1.04–1.08). Frail diabetes participants also used significantly more healthcare resources, assessed as higher likelihood of hospitalization (for 1, 2, and ≥ 3 components, HR 1.06, 95% CI 1.05–1.07; HR 1.16, 95% CI 1.14–1.19; and HR 1.25, 95% CI 1.18–1.33, respectively) and ICU admission (for 1, 2, and ≥ 3 components, HR 1.05, 95% CI 1.03–1.07; HR 1.13, 95% CI 1.08–1.14; and HR 1.17, 95% CI 1.06–1.28, respectively), than non-frail ones (Table 4). Cox proportional hazard-based survival and event-free curves for overall survival, cardiovascular events, hospitalization, and ICU admission dictated essentially dictated similar findings (Fig. 3).

**Table 4:** Adverse outcomes and healthcare utilization among diabetic participants with different severities of frailty

Variables	Number of events	Person-year	Incidence density^a^	Crude HR		Model^b^		Model^c^		Model^d^	
				HR	95% CI	HR	95% CI	HR	95% CI	HR	95% CI
Mortality											
Number of frailty components											
0	26,063	1521,110.06	17.13	1	–	1	–	1	–	1	–
1	13,435	363,900.81	36.92	2.16	2.11–2.2^f^	1.05	1.02–1.07^e^	1.05	1.02–1.07^e^	1.05	1.02–1.07^e^
2	3844	46,547.67	82.58	4.83	4.67–4.99^f^	1.13	1.08–1.17^f^	1.13	1.09–1.18^f^	1.13	1.09–1.18^f^
≥ 3	600	3724.94	161.08	9.42	8.69–10.22^f^	1.25	1.15–1.36^e^	1.25	1.15–1.36^e^	1.25	1.15–1.36^e^
Every 1 component increase				2.15	2.13–2.18^f^	1.06	1.04–1.08^f^	1.06	1.04–1.08^f^	1.06	1.04–1.08^f^
Cardiovascular events											
Number of frailty components											
0	28,054	1,465,567.99	19.14	1	–	1	–	1	–	1	–
1	13,358	338,756.78	39.43	2.05	2.01–2.1^f^	1.05	1.02–1.07^e^	1.05	1.03–1.08^f^	1.05	1.03–1.08^f^
2	3422	40,228.14	85.06	4.38	4.22–4.54^f^	1.15	1.1–1.2^f^	1.16	1.1–1.2^f^	1.16	1.1–1.2^f^
≥ 3	375	3083.18	121.63	6.18	5.58–6.85^f^	1.13	1.01–1.25^g^	1.13	1.02–1.25^e^	1.13	1.02–1.25^g^
Every 1 component increase				2.03	2–2.05^f^	1.06	1.04–1.08^f^	1.06	1.04–1.08^f^	1.06	1.04–1.08^f^
Hospitalization											
Number of frailty components											
0	144,963	1,180,101.25	122.84	1	–	1	–	1	–	1	–
1	49,391	249,466.94	197.77	1.58	1.57–1.6^f^	1.06	1.05–1.07^f^	1.06	1.05–1.07^f^	1.06	1.05–1.07^f^
2	9787	24,850.17	393.84	3.01	2.95–3.07^f^	1.16	1.14–1.19^f^	1.16	1.13–1.19^f^	1.16	1.13–1.19^f^
≥ 3	1040	1659.98	626.51	4.59	4.32–4.88^f^	1.25	1.18–1.33^f^	1.25	1.17–1.33^f^	1.25	1.17–1.33^f^
Every 1 component increase				1.65	1.64–1.67^f^	1.07	1.06–1.08^f^	1.07	1.06–1.08^f^	1.07	1.06–1.08^f^
ICU admission											
Number of frailty components											
0	31,913	1,470,810.05	21.7	1	–	1	–	1	–	1	–
1	14,758	342,678.79	43.07	1.98	1.94–2.02^f^	1.05	1.03–1.07^f^	1.05	1.02–1.07^f^	1.05	1.03–1.07^f^
2	3839	41,595.75	92.29	4.21	4.07–4.36^f^	1.13	1.08–1.14^f^	1.13	1.08–1.17^f^	1.13	1.09-1.17^f^
≥ 3	492	3140.36	156.67	7.07	6.47–7.73^f^	1.17	1.06–1.28^e^	1.16	1.06–1.27^e^	1.16	1.06–1.27^e^
Every 1 component increase				2	1.97–2.02^f^	1.06	1.04–1.07^f^	1.06	1.04–1.07^f^	1.06	1.04–1.07^f^

*CI* confidence interval, *HR* hazard ratio, *ICU* intensive care unit

^a^Per 1000 person-year

^b^Adjusted for demographic profiles, comorbidities, aDSCI, and medications

^c^Adjusted for demographic profiles, comorbidities (including obesity, mental illnesses, hypoglycemia history), substance use (smoking and alcohol abuse), aDCSI, and medications

^d^Analysis including both type 1 and type 2 DM patients

^e^*p* < 0.01

^f^*p* < 0.001

^g^*p* < 0.05

We further analyzed the primary and secondary endpoints based on the presence of each frailty component per se, instead of frailty component counts. After adjusting for demographic profiles, all comorbidities, aDCSI, all the medications including outcome-modifying medications and OADs, and other frailty components, we found that Fatigue (HR 1.03, 95% CI 1–1.06), Resistance (HR 1.21, 95% CI 1.13–1.3), and Body weight loss (HR 1.37, 95% CI 1.3–1.44) were independently associated with higher mortality (Table 5). For cardiovascular events, resistance (HR 1.09, 95% CI 1–1.17), illness (HR 1.17, 95% CI 1.14–1.21), and body weight loss (HR 1.15, 95% CI 1.07–1.23) exhibited risk association. For healthcare utilization, the presence of each of the five components was associated with a significantly higher risk of hospitalization, while impairment in Resistance (HR 1.2, 95% CI 1.12–1.28) and Body weight loss (HR 1.32, 95% CI 1.25–1.4) were associated with a higher risk of ICU admission (Table 5).

**Table 5:** Risk of adverse outcomes and healthcare utilization according to frailty component individually

Variables	Crude		Model^a^	
	HR	95% CI	HR	95% CI
Mortality				
Fatigue	1.3	1.27–1.34^b^	1.03	1.00–1.06^c^
Resistance	2.24	2.09–2.4^b^	1.21	1.13–1.3^b^
Ambulation	3.47	3.22–3.75^b^	1.06	0.98–1.15
Illness	4.52	4.42–4.61^b^	0.99	0.96–1.02
Body weight loss	4.03	3.83–4.23^b^	1.37	1.3–1.44^b^
Cardiovascular events				
Fatigue	1.21	1.18–1.24^b^	0.99	0.96–1.01
Resistance	1.75	1.62–1.9^b^	1.09	1.00–1.17^c^
Ambulation	2.69	2.47–2.94^b^	0.97	0.89–1.07
Illness	4.68	4.58–4.78^b^	1.17	1.14–1.21^b^
Body weight loss	2.21	2.07–2.36^b^	1.15	1.07–1.23^b^
Hospitalization				
Fatigue	1.25	1.24–1.26^b^	1.04	1.03–1.06^b^
Resistance	1.71	1.64–1.77^b^	1.17	1.13–1.22^b^
Ambulation	2.33	2.22–2.44^b^	1.13	1.08–1.19^b^
Illness	2.82	2.79–2.86^b^	1.06	1.04–1.08^b^
Body weight loss	2.09	2.02–2.16^b^	1.22	1.18–1.26^b^
ICU admission				
Fatigue	1.27	1.24–1.3^b^	1.01	0.99–1.04
Resistance	2.04	1.9–2.18^b^	1.2	1.12–1.28^b^
Ambulation	3.08	2.85–3.33^b^	1.07	0.99–1.16
Illness	4.06	3.97–4.14^b^	1.03	1.00–1.06
Body weight loss	3.03	2.87–3.19^b^	1.32	1.25–1.4^b^

*CI* confidence interval, *HR* hazard ratio, *ICU* intensive care unit

^a^Adjusted for demographic profiles, comorbidities, aDSCI, and medications

^b^*p* < 0.001

^c^*p* < 0.05

**Fig. 4:**
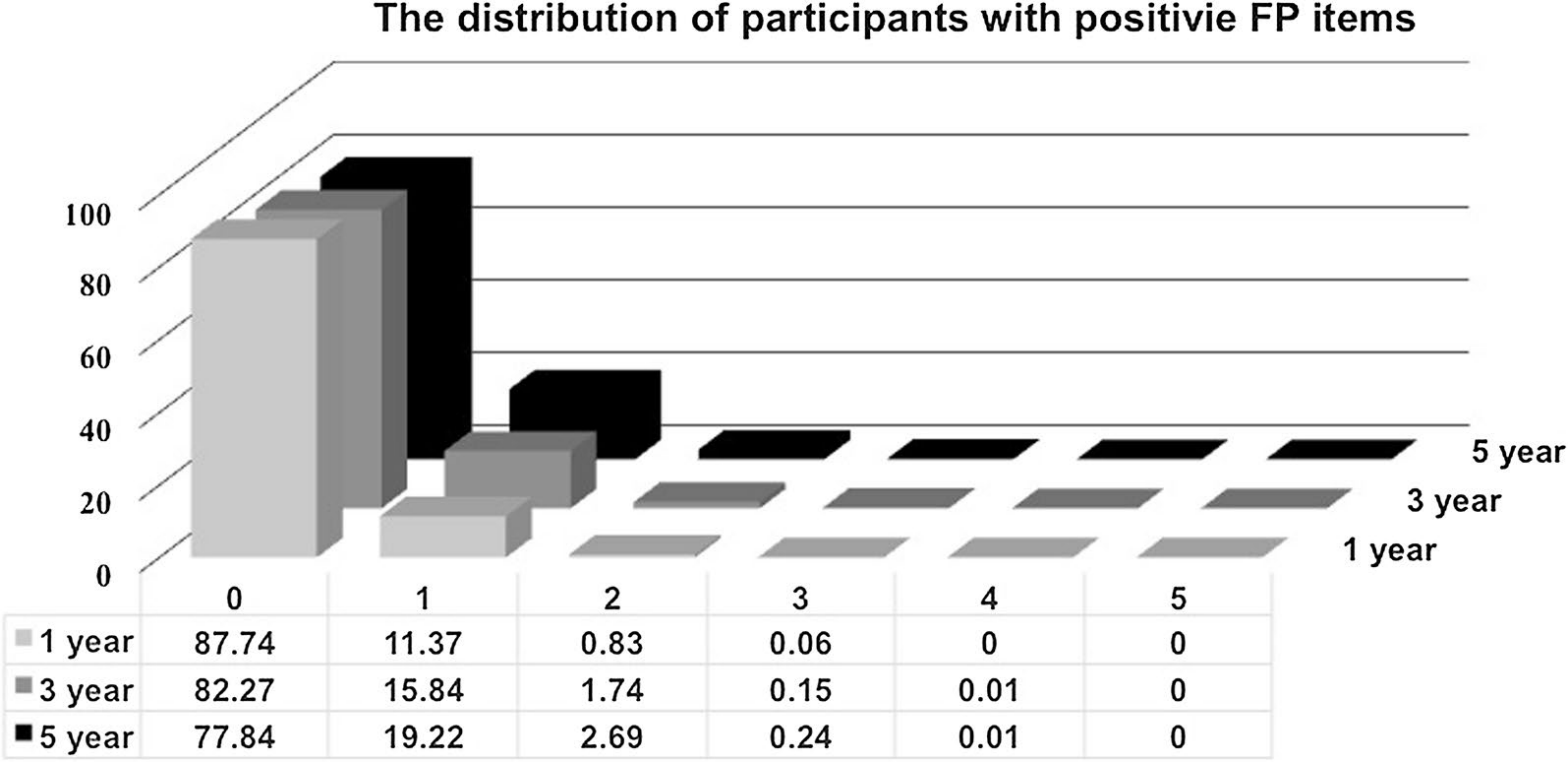
Bar chart of diabetes participants identified with different frailty components, using different lengths of frailty definition. *FP* frail phenotype

Finally, we performed sensitivity analyses by accounting for additional comorbidities and lifestyle factors, varying the length during which frailty components were ascertained (Fig. 4), and including those with type 1 DM (Table 4). After adjusting for mental illnesses history, obesity, hypoglycaemia history, and substance use history, we showed that frail diabetes participants with 1, 2, or ≥ 3 frailty components still had higher overall mortality (for 1, 2, and ≥ 3 components, HR 1.05, 95% CI 1.02–1.07; HR 1.13, 95% CI 1.09–1.18; and HR 1.25, 95% CI 1.15–1.36, respectively) than non-frail ones (Table 4). Similarly, frail diabetes participants with 1, 2, or ≥ 3 frailty components had significantly higher risk of developing cardiovascular events (for 1, 2, and ≥ 3 components, HR 1.05, 95% CI 1.03–1.08; HR 1.16, 95% CI 1.1–1.2; and HR 1.13, 95% CI 1.02–1.25, respectively) than non-frail ones. Frail diabetes participants consumed significantly more healthcare resources, assessed as higher likelihood of hospitalization (for 1, 2, and ≥ 3 components, HR 1.06, 95% CI 1.05–1.07; HR 1.16, 95% CI 1.13–1.19; and HR 1.25, 95% CI 1.17–1.33, respectively) and ICU admission (for 1, 2, and ≥ 3 components, HR 1.05, 95% CI 1.02–1.07; HR 1.13, 95% CI 1.08–1.17; and HR 1.16, 95% CI 1.06–1.27, respectively), than non-frail ones (Table 4). In addition, we used stricter criteria to identify frailty in these diabetes participants through narrowing the time frame of frailty definition from 5 to 1 or 3 years. Pre-frail and frail patients were still associated with a significantly higher risk of mortality, developing cardiovascular events, and utilization of more healthcare resources, compared to non-frail individuals, even after varying the time frame of frailty definition to 1 or 3 years (Additional file 1: Table S1). Finally, the inclusion of type 1 DM patients in our analysis did not alter our findings (Table 4).

## Discussion

In the current study, we estimated the prevalence of pre-frailty and frailty in a nationally representative sample of diabetes patients, based on a widely used FRAIL scale applicable to populations with various diseases. We discovered that patients with pre-frailty and frailty had a significantly higher healthcare utilization and an increased risk of adverse overall and cardiovascular outcome than non-frail individuals; furthermore, this risk increase was correlated with frailty severity independent of other interfering factors, with a 6–7% increase per frailty component. In light of the importance of frailty and also pre-frailty, we propose that early management targeting the entire spectrum of frailty may have the potential to reduce healthcare spending, and thus to benefit public health, in addition to its known effect on patient survival.

Identifying frailty using large claim datasets has become an attractive approach recently [27], because patients with frailty are often under-represented in most clinical studies and claim datasets possess the advantage of large case numbers with a higher probability of balanced clinical features. Using data from Medicare beneficiaries ≥ 65 years old, Kim et al. created a claims-based frailty index approximating a survey-based one, and their results exhibited similar to even better predictive efficacy for adverse health outcomes [37]. A similar approach has been attempted by others using the deficit accumulation approach based on diagnoses groups, with good outcome discrimination ability [38]. On the other hand, Segal et al. implemented Fried’s frail phenotype based on Medicare claims from the Cardiovascular Health Study cohort; similarly, their results were predictive of patient mortality and the risk of nursing home admission [26]. In this study, we adopted a method similar to that of Segal et al. albeit with modifications, including the matching of each included diagnosis to the FRAIL scale (fatigue, resistance, ambulation, illnesses, and weight loss), the exclusion of demographic and socioeconomic variables to mimic the physical frail phenotype directly, and the removal of codes with indirect relationship to the FRAIL construct. Because the results from our approach exhibited excellent predictive efficacy for adverse outcomes (Table 4), we believe that the groups of patients being identified likely have physical frailty with impaired prognosis.

In this study, we found that “Fatigue” was the predominant frailty component in type 2 diabetes patients with pre-frailty and frailty, followed by “Illness” (multimorbidity) (Table 2). This may seem different from the estimated prevalence of each component in the initial FRAIL study (fatigue, 20.1%; illness, 2.1%) [39], however in light of the diabetes background and the advanced age of our frail participants (57.8 vs. 75 years), an increase in multimorbidity prevalence can be reasonable in this study. In addition, studies have demonstrated that diabetes patients had a significantly higher risk of developing chronic fatigue than matched controls, and those with fatigue tend to have more severe functional limitations [40]. The association between hyperglycemia and fatigue may be explained by the co-existence of other diabetic symptoms, psychological distress from self-care, or glucose control variability [41, 42].

During the analysis of associations between each frailty component and outcomes, we found that only “Body weight loss” and “Resistance” impairment were consistently associated with adverse health outcomes and more healthcare utilization, while “Ambulation” was unrelated to adverse health outcomes (Table 5). The low number of diagnoses used to recognize impairment in “Ambulation” and the low prevalence of this frailty component might be plausible reasons to explain these findings. Alternatively, the constituents of healthcare utilization in this study are based more on acute care instead of subacute or long-term care, and that difficulty in ambulation may have a comparatively more prominent association with care types of the latter [43]. Nonetheless, it is evident that each frailty component influences either health outcomes or healthcare utilization.

Past studies that linked healthcare utilization and frailty mostly focused on generally older populations. Comans et al., using a community-based post-acute care cohort, identified that a higher degree of frailty predicted a higher likelihood of re-hospitalization and greater healthcare costs within 6 months, independent of other confounders [44]. In community-dwelling middle-aged to older adults, Blodgett et al. similarly found that frail index scores positively correlated with the risk of hospitalization, more physician visits and medications used [45]. In contrast, the influence of pre-frailty on healthcare utilization is rarely addressed in the literature. A large cross-sectional study in an Australian older population revealed a gradient effect between frailty severity and both hospital and non-hospital based services use [46], providing evidence for the influence of pre-frailty on healthcare utilization. However, a recent study discovered that developing pre-frailty was not associated with an increase in total healthcare utilization in a German cohort [47], rendering this relationship controversial. In the current study, we reported that the status of pre-frailty and frailty were both associated with higher healthcare utilization compared to non-frail patients with DM, in addition to their influence on patient survival. It is plausible that the differences in patient background illness severity account for this discrepancy, because we specifically focused on patients with DM while prior studies addressed older adults in general. It may be easier to detect meaningful differences in populations with higher incidence of outcomes of interest.

Based on our findings, the presence of frailty and even pre-frailty can lead to a higher risk of adverse health outcomes and greater healthcare utilization. Assessing frailty can assist in predicting patient outcomes and in allocating healthcare resource [48]. Moreover, interventions aiming to prevent or ameliorate frailty can offer the chance to reduce healthcare consumption. A recent study suggests that institution-wide frailty assessment can provide valuable information when allocating limited healthcare resources to nursing homes [49], and similar utility is likely demonstrable in patients with DM. Furthermore, care coordination and integration into routine practice has been shown to reduce the frequency of medical encounter among frail elderly, including unplanned hospitalization and medication costs [50]. There are also on-going studies examining the effect of individual-level frailty intervention on healthcare utilization [51]. We believe that frailty-targeted managements can be an important yet under-recognized approach for reducing healthcare resource consumption in DM patients, although further study is still needed for confirmation.

Prior studies reported that the prevalence of frailty in diabetes patients varied between 1 and 48% [11, 12, 52]; in our study, the prevalence of frailty was estimated at 0.3%, which was relatively lower than that reported by others. We propose that under-coding of the diagnoses allocated to the frailty components by physicians [53], the stricter criteria we imposed to identify frailty, and the adoption of the physical frail approach instead of the frail index, might partially account for this discrepancy, although more data are needed to support this conclusion. In addition, we enrolled incident type 2 DM patients instead of long-standing diabetic patients in this study (Fig. 1), and this may be another reason for the low frailty prevalence in our diabetic participants. Furthermore, the mean aDCSI score of our participants was 0.28, significantly lower than that of prevalent DM patients in Taiwan reported previously [18], lending support to the relatively minor diabetic severity and potentially lower prevalence of frailty among these patients.

Our study has its strengths and limitations. The large number of participants enrolled and the comprehensiveness of confounding variables included provide support to the credibility of our findings. Since we did not find any population-based study addressing the influence of frailty on healthcare utilization of diabetic patients, we believe that a study supporting the existence of such association in this population is important to fill the knowledge gap. We also showed that pre-frailty exhibited a similar association with higher healthcare utilization in addition to mortality in diabetes patients, a question rarely addressed before, and our results are expected to clarify the role of pre-frailty in the management of patients with DM. A more in-depth comparison between our findings and those of others shows that there can be differences in the magnitude of risk elevation conferred by frailty between diabetic patients and those with other illnesses (ex. chronic obstructive pulmonary disease or heart failure). These issues warrant further investigation in the future. However, our study is limited by the lack of data from actual physical examinations including vial signs. The claim database in use did not contain information regarding laboratory profiles such as glycated hemoglobin, serum cholesterol or triglyceride levels, and obtaining an accurate history of smoking, alcohol consumption, or comorbidities with a low incidence was difficult. There could be important variables that failed to be considered during our analyses [54–56]. Most importantly, the definition of frailty was based on diagnostic groupings rather than a questionnaire survey, and this approach could suffer from a low sensitivity for detecting abnormalities. As an extended period was required for ascertaining the diagnosis presence in this study, it would be difficult to validate this frailty definition using physical assessment results, since the timing of physical assessment could not be determined. The reported prevalence of each frailty component might not be generalizable to other diabetic population, and extrapolation of our findings should be cautious. Although our findings should be independently confirmed, we believe that programs for early management of frailty and even pre-frailty might be beneficial to diabetes patients in the future [57].

## Conclusion

Using a large representative cohort of patients with type 2 DM, we found that both pre-frailty and frailty increased the risk of long-term mortality and cardiovascular events, and significantly increased healthcare utilization compared to non-frail ones. We believe that management directed against pre-frailty and frailty can reduce healthcare spending in these patients, in addition to improving patient survival.

## Additional file

**Additional file 1: Table S1.** Sensitivity analyses consisting of different ranges of data for identifying frailty.

## Abbreviations

aDCSI: adjusted diabetic complication severity index
ADL: activities of daily living
CHS: Cardiovascular Health Study
CI: confidence interval
DM: diabetes mellitus
HR: hazard ratio
ICD-9-CM: International Classification of Disease-9-Clinical Modification
ICU: intensive care unit
LHDB: Longitudinal Cohort of Diabetes Patients database
OAD: oral anti-diabetic agents
