# Supplementary material for: Both pre-frailty and frailty increase healthcare utilization and adverse health outcomes in patients with type 2 diabetes mellitus
Source: Cardiovasc Diabetol. 2018 Sep 27;17:130. doi: 10.1186/s12933-018-0772-2 (PMC6158921; doi:10.1186/s12933-018-0772-2)
Supplement: Supplementary file 1 — Additional file 1: Table S1. Sensitivity analyses consisting of different ranges of data for identifying frailty. [file 12933_2018_772_MOESM1_ESM.doc]

**Additional file 1: Table S1**. Sensitivity analyses consisting of different ranges of data for identifying frailty

| **Variables** | **Number of events** | **Person-year** | **Incidence density^a^** | **Crude HR** | | **Model^b^** | |
| --- | --- | --- | --- | --- | --- | --- | --- |
|  |  |  |  | **HR** | **95% CI** | **HR** | **95% CI** |
| *Using preceding 3 years of DM diagnosis* | |  |  |  |  |  |  |
| *Mortality* | |  |  |  |  |  |  |
| Number of frailty components |  |  |  |  |  |  |  |
| 0 | 28,610 | 1,605,211.72 | 17.82 | 1 | – | 1 | – |
| 1 | 12,090 | 298,551.38 | 40.5 | 2.27 | 2.23–2.32^c^ | 1.05 | 1.03–1.08^c^ |
| 2 | 2,843 | 29,262.31 | 97.16 | 5.46 | 5.25–5.67^c^ | 1.16 | 1.11–1.21^d^ |
| ≥ 3 | 399 | 2,258.05 | 176.7 | 9.94 | 9.00–10.97^c^ | 1.15 | 1.04–1.27^d^ |
| Every 1 component increase |  |  |  | 2.27 | 2.24–2.3^c^ | 1.06 | 1.04–1.08^c^ |
| *Cardiovascular events* | |  |  |  |  |  |  |
| Number of frailty components |  |  |  |  |  |  |  |
| 0 | 30,869 | 1,544,307.67 | 19.99 | 1 | – | 1 | – |
| 1 | 11,649 | 276,652.3 | 42.11 | 2.1 | 2.05–2.14^c^ | 1.07 | 1.05–1.1^c^ |
| 2 | 2,445 | 24,872.25 | 98.3 | 4.83 | 4.64–5.03^c^ | 1.21 | 1.16–1.27^c^ |
| ≥ 3 | 246 | 1,803.87 | 136.37 | 6.62 | 5.84–7.51^c^ | 1.13 | 0.99–1.28 |
| Every 1 component increase |  |  |  | 2.1 | 2.07–2.13^c^ | 1.08 | 1.06–1.1^c^ |
| *Hospitalization* | |  |  |  |  |  |  |
| Number of frailty components |  |  |  |  |  |  |  |
| 0 | 156,130 | 1,238,294.32 | 126.08 | 1 | – | 1 | – |
| 1 | 41,764 | 201,949.25 | 206.8 | 1.61 | 1.59–1.63^c^ | 1.07 | 1.06–1.08^c^ |
| 2 | 6,618 | 14,939.54 | 442.99 | 3.27 | 3.19–3.35^c^ | 1.18 | 1.15–1.22^c^ |
| ≥ 3 | 669 | 895.23 | 747.29 | 5.22 | 4.84–5.63^c^ | 1.28 | 1.19–1.39^c^ |
| Every 1 component increase |  |  |  | 1.69 | 1.68–1.71^c^ | 1.08 | 1.07–1.09^c^ |
| *ICU admission* |  |  |  |  |  |  |  |
| Number of frailty components |  |  |  |  |  |  |  |
| 0 | 34,969 | 1,550,297.52 | 22.56 | 1 | – | 1 | – |
| 1 | 12,937 | 280,263.99 | 46.16 | 2.04 | 2.00–2.08^c^ | 1.05 | 1.03–1.08^c^ |
| 2 | 2,763 | 25,804.2 | 107.08 | 4.69 | 4.51–4.88^c^ | 1.16 | 1.11–1.21^c^ |
| ≥ 3 | 333 | 1,859.25 | 179.1 | 7.76 | 6.97–8.65^c^ | 1.13 | 1.01–1.26^e^ |
| Every 1 component increase |  |  |  | 2.08 | 2.05–2.11^c^ | 1.06 | 1.04–1.08^c^ |
| *Using preceding 1 years of DM diagnosis* | |  |  |  |  |  |  |
| *Mortality* | |  |  |  |  |  |  |
| Number of frailty components |  |  |  |  |  |  |  |
| 0 | 32,530 | 1,709,824.16 | 19.03 | 1 | – | 1 | – |
| 1 | 9,665 | 211,284.13 | 45.74 | 2.41 | 2.35–2.46^c^ | 1.06 | 1.03–1.08^c^ |
| 2 | 1,572 | 13,303.04 | 118.17 | 6.22 | 5.91–6.54^c^ | 1.11 | 1.05–1.17^d^ |
| ≥ 3 | 175 | 872.15 | 200.65 | 10.6 | 9.12–12.27^c^ | 1.04 | 0.9–1.21 |
| Every 1 component increase |  |  |  | 2.4 | 2.37–2.45^c^ | 1.05 | 1.03–1.07^c^ |
| *Cardiovascular events* | |  |  |  |  |  |  |
| Number of frailty components |  |  |  |  |  |  |  |
| 0 | 34,960 | 1,640,875.58 | 21.31 | 1 | – | 1 | – |
| 1 | 8,860 | 195,018.87 | 45.43 | 2.12 | 2.07–2.17^c^ | 1.09 | 1.06–1.12^c^ |
| 2 | 1,277 | 11,084.03 | 115.21 | 5.29 | 5.00–5.59^c^ | 1.2 | 1.13–1.27^c^ |
| ≥ 3 | 112 | 657.62 | 170.31 | 7.68 | 6.38–9.24^c^ | 1.18 | 0.98–1.42 |
| Every 1 component increase |  |  |  | 2.17 | 2.13–2.2^c^ | 1.09 | 1.07–1.11^c^ |
| *Hospitalization* | |  |  |  |  |  |  |
| Number of frailty components |  |  |  |  |  |  |  |
| 0 | 171,054 | 1,307,685.43 | 130.81 | 1 | – | 1 | – |
| 1 | 30,556 | 141,696.33 | 215.64 | 1.62 | 1.60–1.64^c^ | 1.07 | 1.06–1.09^c^ |
| 2 | 3,293 | 6,376.3 | 516.44 | 3.62 | 3.50–3.75^c^ | 1.21 | 1.16–1.25^c^ |
| ≥ 3 | 278 | 320.28 | 867.99 | 5.78 | 5.15–6.50^c^ | 1.23 | 1.09–1.39^d^ |
| Every 1 component increase |  |  |  | 1.7 | 1.69–1.72^c^ | 1.08 | 1.07–1.09^c^ |
| *ICU admission* |  |  |  |  |  |  |  |
| Number of frailty components |  |  |  |  |  |  |  |
| 0 | 39,425 | 1,648,382.79 | 23.92 | 1 | – | 1 | – |
| 1 | 9,976 | 197,637.33 | 50.48 | 2.1 | 2.06–2.15^c^ | 1.05 | 1.03–1.08^c^ |
| 2 | 1,454 | 11,505.04 | 126.38 | 5.2 | 4.94–5.48^c^ | 1.11 | 1.05–1.17^d^ |
| ≥ 3 | 357 | 699.79 | 210.06 | 8.52 | 7.25–10.02^c^ | 1.05 | 0.89–1.24 |
| Every 1 component increase |  |  |  | 2.16 | 2.12–2.19^c^ | 1.05 | 1.03–1.07^c^ |

^a^ per 1000 person-year

^b^ Adjusted for demographic profiles, comorbidities, aDSCI, and medications

^c^ *p* < 0.001

^d^ *p* < 0.01

^e^ *p* < 0.05

CI, confidence interval; DM, diabetes mellitus; HR, hazard ratio; ICU, intensive care unit
